# Genetics of Host Response to *Leishmania tropica* in Mice – Different Control of Skin Pathology, Chemokine Reaction, and Invasion into Spleen and Liver

**DOI:** 10.1371/journal.pntd.0001667

**Published:** 2012-06-05

**Authors:** Tetyana Kobets, Helena Havelková, Igor Grekov, Valeriya Volkova, Jarmila Vojtíšková, Martina Slapničková, Iryna Kurey, Yahya Sohrabi, Milena Svobodová, Peter Demant, Marie Lipoldová

**Affiliations:** 1 Laboratory of Molecular and Cellular Immunology, Institute of Molecular Genetics, Academy of Sciences of the Czech Republic, Prague, Czech Republic; 2 Faculty of Science, Charles University, Prague, Czech Republic; 3 Roswell Park Cancer Institute, Buffalo, New York, United States of America; Queensland Institute of Medical Research, Australia

## Abstract

**Background:**

Leishmaniasis is a disease caused by protozoan parasites of genus *Leishmania*. The frequent involvement of *Leishmania tropica* in human leishmaniasis has been recognized only recently. Similarly as *L. major*, *L. tropica* causes cutaneous leishmaniasis in humans, but can also visceralize and cause systemic illness. The relationship between the host genotype and disease manifestations is poorly understood because there were no suitable animal models.

**Methods:**

We studied susceptibility to *L. tropica*, using BALB/c-c-STS/A (CcS/Dem) recombinant congenic (RC) strains, which differ greatly in susceptibility to *L. major*. Mice were infected with *L. tropica* and skin lesions, cytokine and chemokine levels in serum, and parasite numbers in organs were measured.

**Principal Findings:**

Females of BALB/c and several RC strains developed skin lesions. In some strains parasites visceralized and were detected in spleen and liver. Importantly, the strain distribution pattern of symptoms caused by *L. tropica* was different from that observed after *L. major* infection. Moreover, sex differently influenced infection with *L. tropica* and *L. major*. *L. major-*infected males exhibited either higher or similar skin pathology as females, whereas *L. tropica*-infected females were more susceptible than males. The majority of *L. tropica*-infected strains exhibited increased levels of chemokines CCL2, CCL3 and CCL5. CcS-16 females, which developed the largest lesions, exhibited a unique systemic chemokine reaction, characterized by additional transient early peaks of CCL3 and CCL5, which were not present in CcS-16 males nor in any other strain.

**Conclusion:**

Comparison of *L. tropica* and *L. major* infections indicates that the strain patterns of response are species-specific, with different sex effects and largely different host susceptibility genes.

## Author Summary

Several hundred million people are exposed to the risk of leishmaniasis, a disease caused by intracellular protozoan parasites of several *Leishmania* species and transmitted by phlebotomine sand flies. In humans, *L. tropica* causes cutaneous form of leishmaniasis with painful and long-persisting lesions in the site of the insect bite, but the parasites can also penetrate to internal organs. The relationship between the host genes and development of the disease was demonstrated for numerous infectious diseases. However, the search for susceptibility genes in the human population could be a difficult task. In such cases, animal models may help to discover the role of different genes in interactions between the parasite and the host. Unfortunately, the literature contains only a few publications about the use of animals for *L. tropica* studies. Here, we report an animal model suitable for genetic, pathological and drug studies in *L. tropica* infection. We show how the host genotype influences different disease symptoms: skin lesions, parasite dissemination to the lymph nodes, spleen and liver, and increase of levels of chemokines CCL2, CCL3 and CCL5 in serum.

## Introduction

Several hundred million people in 88 countries are living in areas where they can contract leishmaniasis, a disease caused by intracellular protozoan parasites of the genus *Leishmania* and transmitted to vertebrates by phlebotomine sand flies. *Leishmania* parasites infect professional phagocytes (neutrophils, monocytes and macrophages), as well as dendritic cells and fibroblasts [1]. The main vertebrate host target cell is the macrophage, where parasites multiply, eventually rupture the cell, and spread to uninfected cells [2]. As macrophages migrate to all mammalian tissues, *Leishmania* parasites have a great potential for damaging bodily functions. In the dermis, they cause the cutaneous form of the disease (which can be localized or diffuse); in the mucosa, they result in mucocutaneous leishmaniasis; and the metastatic spread of infection to the spleen and liver leads to visceral leishmaniasis. One of the major factors determining the type of pathology is the species of *Leishmania* [3]. However, the transmitting vector, as well as genotype, nutritional status of the host, and environmental and social factors also have a large impact on the outcome of the disease [3], [4]. That is why even patients, infected by the same species of *Leishmania*, develop different symptoms [3] and may differ in their response to therapy [5]. The basis of this heterogeneity is not well understood [6], but part of this variation is likely genetic. Numerous potentially relevant genes were reported (reviewed in [7]).

The extent of involvement of *Leishmania tropica* in human leishmaniasis has been recognized only recently. The western limit of *L. tropica* appears to be the Greek Island of Zakynthos, the disease has been found in Turkey, Syria, Jordan, Israel, Morocco, Tunisia, Saudi Arabia, Yemen, Iran, Iraq, Afghanistan, Turkmenistan, Pakistan, Kenya, Ethiopia and Namibia, and at its eastern limits in India (reviewed in [8]). While *L. major* is a zoonosis with mainly rodent (*Gerbillidae*) reservoir hosts, *L. tropica* can circulate among humans without the involvement of animal reservoirs, but zoonotic transmission also occurs [9]. Similarly to *L. major*, *L. tropica* causes cutaneous leishmaniasis in humans. Moreover, *L. tropica* was also reported to visceralize and cause an initially not understood systemic illness in veterans returning from endemic areas in the Middle East [10], as well as the classical visceral leishmaniasis (kala-azar) in India [11], and the disseminated cutaneous leishmaniasis accompanied with visceral leishmaniasis in Iran [12].

A suitable animal model for study of this parasite would contribute to functional dissection of the clinical course of infection. Golden hamsters (*Mesocricetus auratus*) have been considered to be the best model host of the *L. tropica* infection, but this host is not inbred and hence not suitable for many studies. However, several strains of *L. tropica* from Afghanistan, India [13], and Turkey [14] have been described to cause cutaneous disease in inbred BALB/c mice, thus providing a better defined host. In comparison with widely studied immune response to *L. major* infection (reviewed in [15]) and its genetic control (reviewed in [7]), little is known about *L. tropica* in mouse [13], [14], [16]. Here we aimed to study genetics of susceptibility to *L. tropica* in the mouse. We analyzed response to *L. tropica* in CcS/Dem (CcS) recombinant congenic (RC) strains [17] derived from the background strain BALB/cHeA (BALB/c) and the donor strain STS. Each CcS strain contains a different unique random set of about 12.5% genes from the donor strain STS and 87.5% genes from the background strain BALB/c. These strains have been already successfully used for analysis of infection with *Leishmania major* [18]–[22]. The RC system enabled us to analyze organ pathology and systemic disease after infection with *L. tropica* and their genetic control.

## Materials and Methods

### Mice

Males and females of strains BALB/c, STS and selected RC strains [17], [23] were tested. When used for these experiments, RC strains were in more than 38 generation of inbreeding and therefore highly homozygous. During the experiment, male and female mice were placed into separate rooms and males were caged individually. The research had complied with all relevant European Union guidelines for work with animals and was approved by the Institutional Animal Care Committee of the Institute of Molecular Genetics AS CR and by Departmental Expert Committee for the Approval of Projects of Experiments on Animals of the Academy of Sciences of the Czech Republic.

#### Experiments with *Leishmania tropica*

Mice of the strains BALB/c (12 females, 12 males), STS (12 females, 13 males), CcS-3 (12 females, 10 males), CcS-5 (11 females, 12 males), CcS-11 (14 females, 19 males), CcS-12 (6 females, 6 males), CcS-16 (12 females, 12 males), CcS-18 (8 females, 12 males), and CcS-20 (12 females, 12 males) were infected with *L. tropica*. Mice were tested in 2 successive experimental groups and were killed 21 and 43 weeks after infection. The age of the mice at the time of infection was 9 to 26 weeks (mean 16 weeks, median 16 weeks).

#### Experiments with *Leishmania major*

Mice of the strains BALB/c (27 females, 30 males), STS (8 females, 10 males), CcS-3 (10 females, 10 males), CcS-5 (26 females, 32 males), CcS-11 (20 females, 21 males), CcS-12 (18 females, 17 males), CcS-16 (11 females, 13 males), CcS-18 (9 females, 4 males), and CcS-20 (14 females, 18 males) were infected with *L. major*. Mice were tested in 8 successive experimental groups and were killed 8 weeks after infection. The age of the mice at the time of infection was 8 to 47 weeks (mean 16 weeks, median 15 weeks).

### Parasite

*Leishmania tropica* from Urfa, Turkey (MHOM/1999/TR/SU23) was used for infecting mice. Amastigotes were transformed to promastigotes using SNB-9 [24], 1×10^7^ stationary phase promastigotes from subculture 2 have been inoculated in 50 µl of sterile saline s.c. into the tail base, with promastigote secretory gel (PSG) collected from the midgut of *L. tropica*-infected *Phlebotomus sergenti* females (laboratory colony originating from *L. tropica* focus in Urfa). PSG was collected as described [25]. The amount corresponding to one sand fly female was used per mouse.

*Leishmania major* LV 561 (MHOM/IL/67/LRC-L137 JERICHO II) was used for mouse infection. Amastigotes were transformed to promastigotes using SNB-9 [24], 1×10^7^ promastigotes from 6 days old subculture 2 were inoculated in 50 µl of sterile saline s.c. into the tail base.

### Disease phenotype

The size of the skin lesions was measured weekly using a Vernier caliper gauge. The mice infected with *L. tropica* were killed 21 or 43 weeks after inoculation. Mice infected with *L. major* were killed 8 weeks after infection. Blood, spleen, liver and inguinal lymph nodes were collected for further analysis.

### Quantification of parasite load in spleens and inguinal lymph nodes

The current semi-quantitative technique is based on the limiting dilution assay [26]. We modified the procedure by using only a single pre-selected cell concentration, and parasite count was measured with a Coulter Counter CBC5 (Coulter Electronics Inc., Hialeah, FL). In comparison with the original limiting dilution technique, this modified culture method is less laborious and allows rapid estimation of parasite number.

Preparation of cells must be carried out under sterile conditions. During preparation, all samples, which were not immediately worked with, were kept on ice. Inguinal lymph nodes and quarters of spleens were disrupted in a glass homogenizer in complete RPMI (containing 5% of heat-inactivated fetal calf serum (Sigma-Aldrich, USA), 25 mM Hepes (Sigma-Aldrich, USA), 0.0005% β-mercaptoethanol (Serva, Germany), 63.7 µg/ml penicillin (Sigma-Aldrich, USA), and 100 µg/ml streptomycin (Sigma-Aldrich, USA). The homogenate was passed through the nylon filter. The homogenizer was washed 3 times with 3 ml of sterile PBS after processing each lymph node. The samples were then centrifuged 8 min at 300 g, 4°C (centrifuge Eppendorf 5810 R, Eppendorf, Germany). The supernatant was removed and the cells were resuspended in 0.5 ml of complete Schneider's medium supplemented with 20% heat-inactivated fetal calf serum (Sigma-Aldrich, USA), 2% sterile fresh human urine, 50 µg/ml gentamicine (Sigma-Aldrich, USA), 63.7 µg/ml penicillin (Sigma-Aldrich, USA), and 100 µg/ml streptomycin (Sigma-Aldrich, USA). To count cells with a Coulter Counter CBC5 (Coulter Electronics Inc., Hialeah, FL), USA), 50 µl of the cell suspension was diluted in 20 ml of PBS. Ekoglobin (Hemax s.r.o., Czech Republic) was added to the diluent prior to counting to lyse red blood cells.

0.5 ml of the cell suspension (1×10^5^ cells per ml for lymph nodes and 2×10^5^ cells per ml for spleens) was cultivated in complete Schneider's medium in 48-well tissue culture plates (Costar, Corning Inc., USA) at 27°C (Biological thermostat BT 120 M, Labsystem, Finland) for 3 days. Each sample was prepared in triplicate. After incubation, 100 µl of a mixed sample from each well, containing *Leishmania* parasites released from lymph node or spleen cells were diluted in 20 ml of PBS and the parasite number was counted with the Coulter Counter.

### Quantification of parasite load in livers

Parasite load was measured in frozen liver samples using PCR-ELISA according to the previously published protocol [27]. Briefly, total DNA was isolated using a standard proteinase procedure. For PCR, two primers (digoxigenin-labeled F 5′-ATT TTA CAC CAA CCC CCA GTT-3′ and biotin-labeled R 5′-GTG GGG AGG GGG CGT TCT-3′ (VBC Genomics Biosciences Research, Austria) were used for amplification of the 120-bp conservative region of the kinetoplast minicircle of *Leishmania* parasite, and 50 ng of extracted DNA was used per each PCR reaction. For a positive control, 20 ng of *L. tropica* DNA per reaction was amplified as a highest concentration of standard. A 40-cycle PCR reaction was used for detection. Parasite load was determined by analysis of the PCR product with the modified ELISA protocol (Pharmingen, USA). Concentration of *Leishmania* DNA was determined using the ELISA Reader Tecan and the curve fitter program KIM-E (Schoeller Pharma, Czech Republic) with least squares-based linear regression analysis.

### Cytokine and IgE levels

Levels of GM-CSF (granulocyte-macrophage colony-stimulating factor), CCL2 (chemokine ligand 2)/MCP-1 (monocyte chemotactic protein-1), CCL3/MIP-1α (macrophage inflammatory protein-1α), CCL4/MIP-1β (macrophage inflammatory protein-1β), CCL5/RANTES (regulated upon activation, normal T-cell expressed, and secreted) and CCL7/MCP-3 (monocyte chemotactic protein-3) in serum were determined using Mouse chemokine 6-plex kit (Bender MedSystems, Austria). The kit contains two sets of beads of different size internally dyed with different intensities of fluorescent dye. The set of small beads was used for GM-CSF, CCL5/RANTES and CCL4/MIP-1β and the set of large beads for CCL3/MIP-1α, CCL2/MCP-1 and CCL7/MCP-3. The beads are coated with antibodies specifically reacting with each of the analytes (chemokines) to be detected in the multiplex system. A biotin secondary antibody mixture binds to the analytes captured by the first antibody. Streptavidin-phycoerythrin binds to the biotin conjugate and emits a fluorescent signal. The test procedure was performed in the 96 well filter plates (Millipore, USA) according to the protocol of Bender MedSystem. Beads were analyzed on flow cytometer LSR II (BD Biosciences, USA). As standards were used lyophilized GM-CSF and chemokines (CCL2/MCP-1, CCL3/MIP1α, CCL4/MIP1β, CCL5/RANTES, CCL7/MCP-3) supplied in the kit. Concentration was evaluated by Flow Cytomix Pro 2.4 software (eBioscience, Vienna, Austria). The limit of detection of each analyte was determined to be for GM-CSF 12.2 pg/ml, CCL2/MCP-1 42 pg/ml, CCL7/MCP-3 1.4 pg/ml, CCL3/MIP-1α 1.8 pg/ml, CCL4/MIP-1β 14.9 pg/ml, CCL5/RANTES 6.1 pg/ml respectively.

IFNγ, IL-4, IL-12 and IgE levels in serum were determined using the primary and secondary monoclonal antibodies (IFNγ: R4-6A2, XMG1.2; IL-4: 11B11, BVD6-24G2; IL-12p40/p70: C15.6, C17.8; IgE: R35-72, R35118) and standards from BD Biosciences, USA (recombinant mIFNγ, mIL-4, mIL-12 and purified mIgE: C38-2). ELISA was performed as recommended by BD Biosciences. The ELISA measurement of IFNγ, IL-4, IL-12, and IgE levels was performed by the ELISA Reader Tecan and the curve fitter program KIM-E (Schoeller Pharma, Czech Republic) using least squares-based linear regression analysis. The detection limit of ELISA was determined to be 30 pg/ml for IFNγ, 8 ng/ml for IgE, 16 pg/ml for IL-4 and 15 pg/ml for IL-12.

### Staining of tissue sections

Inguinal lymph nodes and spleens were fixed in 4% formaldehyde and embedded in paraffin. Immunohistochemical staining of parasites was performed in 5 µm lymph node sections. Slides were deparaffinized with xylene (2 times for 5 min) and rehydrated with 96% ethanol (3 times for 3 min), 80% ethanol (3 min), 70% ethanol (3 min) and PBS (phosphate buffer saline, 3 min). Endogenous peroxidase was quenched with 3% H_2_O_2_ in methanol for 10 min. Sections were washed in PBS (10 min) and parasites were stained using anti-*Leishmania* lipophosphoglycan monoclonal mouse IgM (Code Nr. CLP003A, Cedarlane, Canada) diluted 1∶100 in PBS with 1% BSA (bovine serum albumine, Sigma-Aldrich, USA) and applied for 1 h at 37°C, followed by TRITC-conjugated goat anti-mouse IgM (Code Nr. 115-025-020, Jackson Immunoresearch, USA), also diluted 1∶100 in PBS with 1% BSA and applied for 1 h at 37°C. Cell nuclei were stained with DAPI (4′,6-diamidino-2-phenylindole dihydrochloride) 10 ng/µl (Sigma-Aldrich, USA). For histological analysis, 5 µm spleen and lymph node sections were stained by the routine hematoxylin and eosin method (H&E).

### Statistical analysis

The differences between CcS/Dem strains in parasite numbers in lymph nodes were evaluated by the analysis of variance (ANOVA) and Newman-Keuls multiple comparison using the program Statistica for Windows 8.0 (StatSoft, Inc., Tulsa, Oklahoma, U.S.A.). Strain and age were fixed factors and individual experiments were considered as a random parameter. The differences in parameters between strains were evaluated using the Newman-Keuls multiple comparison test at 95% significance. Difference between sexes in parasite numbers in lymph nodes was analysed by Mann Whitney U test. Analysis of sex influence on lesion development after *L. major* infection was performed using General Linear Models, Univariate ANOVA, Statistica 8.0 with experiment as a random and age as a fixed parameter.

## Results

### Genetic differences in skin pathology caused by infection with *L. tropica*

To study susceptibility to *L. tropica* we infected both females and males of the strains BALB/c, STS and RC strains CcS-3, CcS-5, CcS-11, CcS-12, CcS-16, CcS-18, and CcS-20.

**Figure 1:**
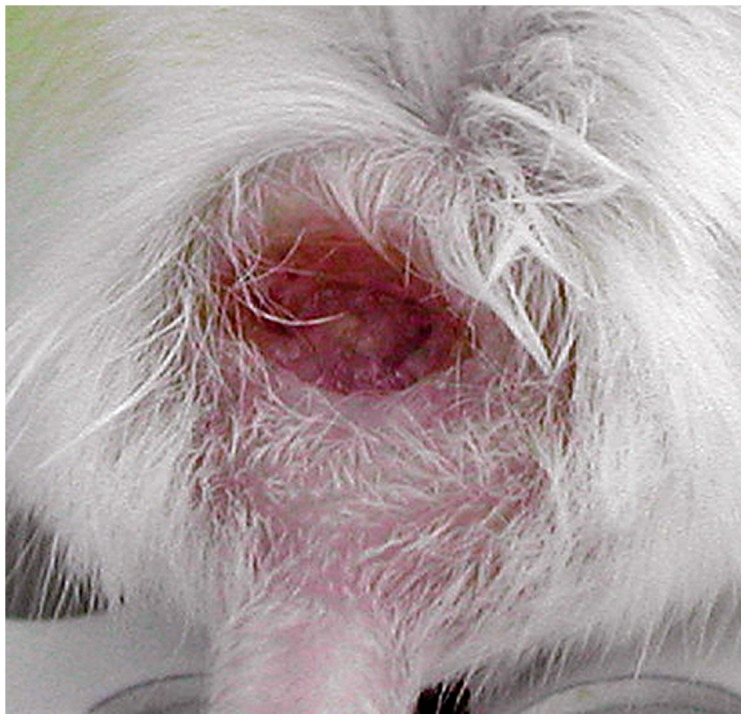
Skin lesion caused by *Leishmania tropica* in female of CcS-16 strain at week 43 after infection.

**Figure 2:**
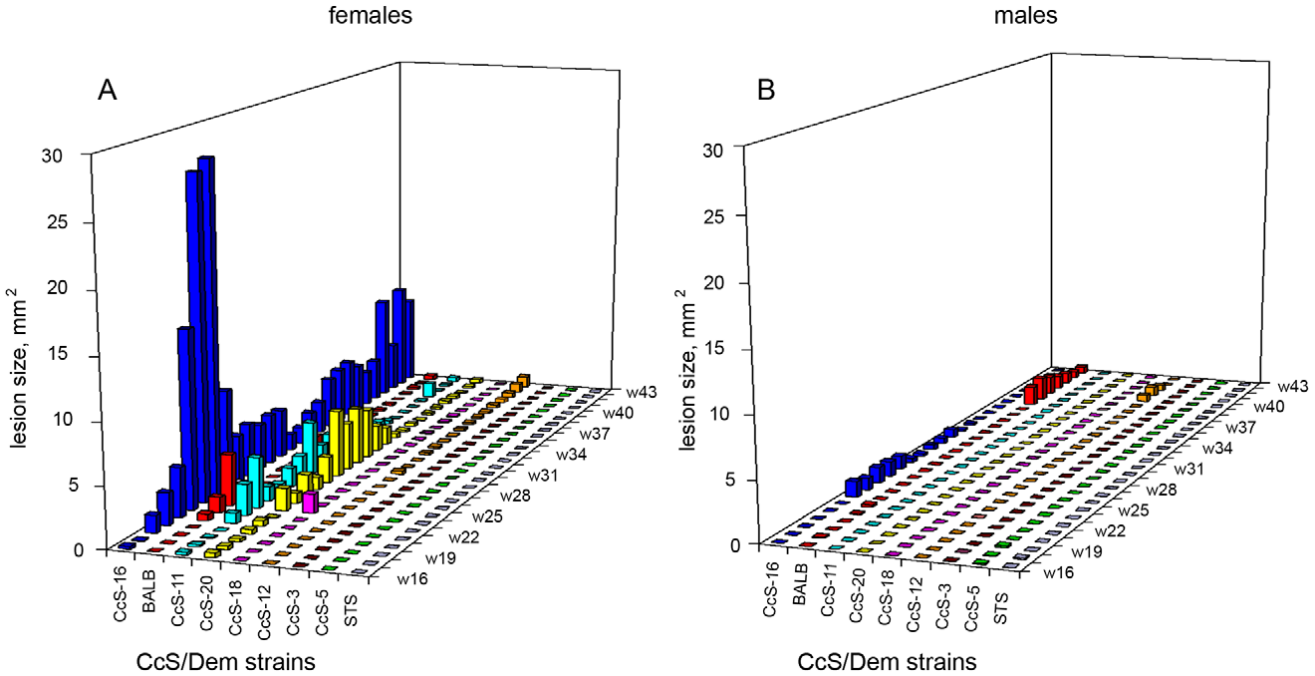
Kinetics of skin lesion development of CcS/Dem strains after infection with *L. tropica*. Individual strains are marked with different colors. The columns show median values of lesion size in females (A) and males (B). Figure summarizes data from two independent experiments.

In females, skin pathology started as a nodule at the site of *L. tropica* infection appearing between weeks 11 and 20, which transformed in susceptible strains into a skin lesion (Figure 1). Females of the strains BALB/c, CcS-11, CcS-16 and CcS-20 were relatively susceptible to the infection and developed skin lesions after week 18; the largest lesions were observed in CcS-16 (Figure 2A). Females of the strain CcS-16 exhibited skin lesions from week 18 until the end of experiment (week 43). In females of the strains BALB/c, CcS-11 and CcS-20, the lesions partly healed and tended to transform back to nodules after week 30. Interestingly, in females of the strain CcS-11, small skin nodules appeared at week 14, but at 32–42 weeks of infection half of the females died without obvious pathological findings. Only one female died at week 13 in the 21-week experiment. Strains CcS-12 and CcS-18 are intermediate in susceptibility to skin pathology. CcS-12 females developed small skin lesions at the late stages of infection (after week 37), whereas CcS-18 females developed nodules or small lesions that healed. Strains STS, CcS-3 and CcS-5 were resistant to skin lesions development. Females of the strain CcS-3 had small skin nodules at the late stages of infection and did not develop skin lesions during the entire course of the experiment. Females of the strains STS and CcS-5 were resistant to *L. tropica*, and only a few of them developed small nodules at the site of infection.

Males of the strain CcS-16 developed small lesions from week 22, which later healed, whereas BALB/c males exhibited small lesions from week 30 until the end of the experiment (Figure 2B). Males of the strain CcS-12 developed small skin lesions at the late stages of infection (after week 37). Males of CcS-11 developed no or only small skin nodules, but most of the animals died before week 18 of infection. Similarly as CcS-11 females, they were without obvious pathological findings. Males of the strains STS, CcS-3 and CcS-5 had small skin nodules at the late stages of infection and did not develop skin lesions within the entire course of the experiment. Only a few males of the strains CcS-18 and CcS-20 developed small nodules at the site of infection.

### Sex has different effect on skin pathology caused by *L. tropica* and *L. major*

Sex differences observed in susceptibility to *L. tropica* led us to analyze sex influence in susceptibility to *L. major*. As our previous research with *L. major* was focused on analysis of females [28], in this study we have infected with *L. major* both females and males of strains BALB/c, STS and RC strains CcS-3, CcS-5, CcS-11, CcS-12, CcS-16, CcS-18, and CcS-20. Both males and females of all analyzed strains developed larger skin lesions after infection with *L. major* than when infected with *L. tropica* (Table 1, Figure 2A, B). The effect of sex was different in experiments with *L. major* and *L. tropica*. After the infection with *L. tropica*, females of CcS/Dem strains in most cases exhibited more extensive skin pathology than males (Figure 2 A, B), whereas after infection with *L. major*, skin lesions in males and females of strains BALB/c, STS, CcS-11, CcS-12, CcS-16 and CcS-20 did not differ, whereas males of strains CcS-3 (*P* = 0.001), CcS-5 (*P* = 0.001) and probably also CcS-18 (*P* = 0.043) developed larger skin lesions than females (Table 1).

**Table 1:** Sex differences in lesion size (week 8) after *L. major* infection.

Strain	Sex	Number of mice	Lesion size week 8 in mm^2^		*P*-level of difference between females and males
			mean	±SD	
BALB/c	females	27	116.7	±30.4	0.098
BALB/c	males	30	144.3	±60.5	
STS	females	8	3.0	±7.4	0.252
STS	males	10	6.9	±8.2	
**CcS-3**	females	10	45.2	±13.5	**0.001**
**CcS-3**	males	10	73.8	±17.2	
**CcS-5**	females	26	14.9	±15.3	**0.001**
**CcS-5**	males	32	29.7	±27.9	
CcS-11	females	20	61.7	±38.1	0.074
CcS-11	males	21	68.6	±44.2	
CcS-12	females	18	134.6	±27.0	0.296
CcS-12	males	17	146.5	±44.4	
CcS-16	females	11	138.0	±32.7	0.105
CcS-16	males	13	160.8	±28.2	
**CcS-18**	females	9	97.5	±36.8	**0.043**
**CcS-18**	males	4	126.2	±38.0	
CcS-20	females	14	51.6	±31.7	0.665
CcS-20	males	18	57.4	±36.0	

Analysis of sex influence on lesion development was performed using General Linear Models Univariate ANOVA with experiment as a random and age as a fixed parameter. We have observed influence of experiment (*P* range 0.006–0.000019), whereas influence of age was not significant (*P*>0.17).

### Draining lymph nodes of all tested strains contained viable *L. tropica* parasites

**Figure 3:**
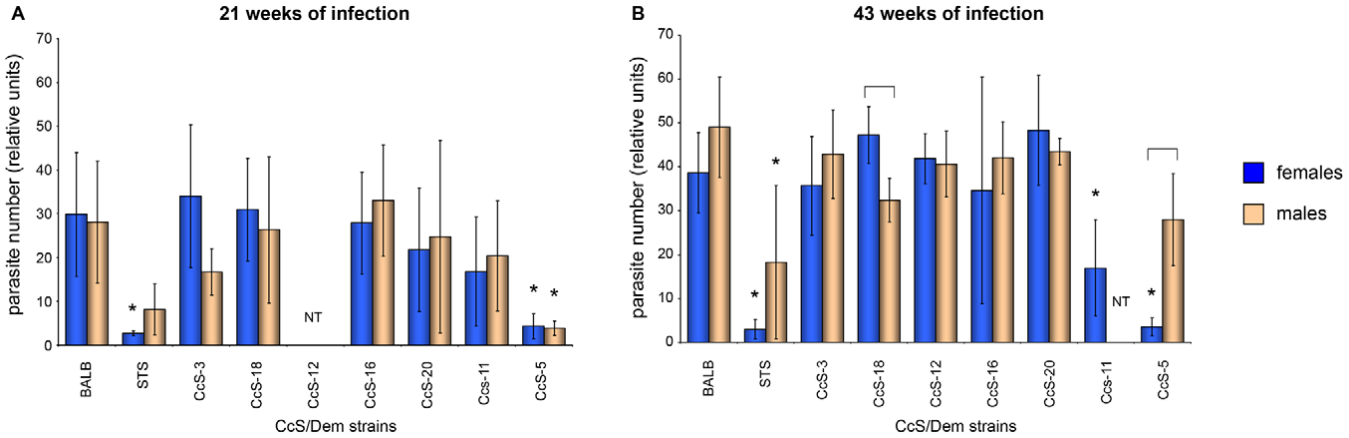
Number of parasites cultivated from lymph nodes of mouse strains 21 and 43 weeks after infection. Mice were killed at week 21 (A) and week 43 (B) after infection. Asterisks show strains that exhibited parasite load significantly different from BALB/c. Brackets indicate strains with differences between males and females. Data are presented as mean ± SD.

**Figure 4:**
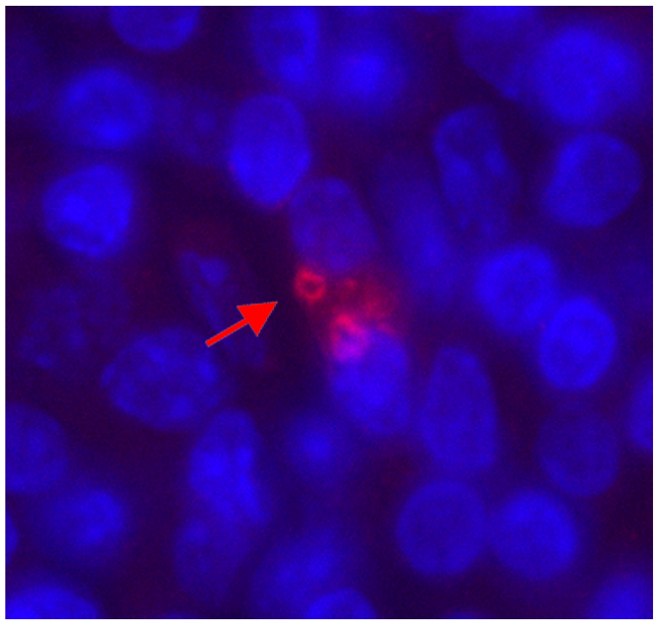
*Leishmania tropica* parasites inside the macrophage. A smear of the inguinal lymph node of BALB/c male was stained with the anti-*Leishmania* lipophosphoglycan monoclonal antibody (CLP003A, Cedarlane, Hornby, Canada) and TRITC labeled IgM (115-025-020, Jackson Immunoresearch, West Grove, PA, United States of America), all diluted 1∶100. Nuclei of the cells were stained with DAPI. Parasites are shown with arrows.

*In vitro* culture tests showed that all tested mice, including strains that did not exhibit any skin pathology, contained viable parasites in their inguinal lymph nodes both 21 and 43 weeks after infection (Figure 3). Presence of parasites was also documented by staining of *Leishmania* in tissue smears with the anti-*Leishmania* lipophosphoglycan monoclonal antibody (Figure 4) and by histological analysis of hematoxylin-eosin stained lymph nodes smears (Figure S1).

None of the strains contained more parasites in the lymph nodes than the background parental strain BALB/c (Figure 3). At week 21 after infection, females of the strains STS and CcS-5 (*P* = 0.0002), and males of the strain CcS-5 (*P* = 0.0093) contained significantly lower parasite numbers than the strain BALB/c. At week 43 after infection, females of the strains STS, CcS-5 and CcS-11 (*P* = 0.00000001), and STS males (*P* = 0.0004) had significantly lower parasite load than BALB/c. At week 43, females of strain CcS-18 had higher parasite count than males (*P* = 0.0209), whereas males of the strain CcS-5 had higher parasite load than females (*P* = 0.0143). In both experiments counted together, females of CcS-18 (*P* = 0.0318) strain had higher parasite load than males, whereas STS males had higher parasite numbers than females (*P* = 0.0097) (Figure 3).

### *L. tropica* can invade spleens and livers of several mouse strains

**Figure 5:**
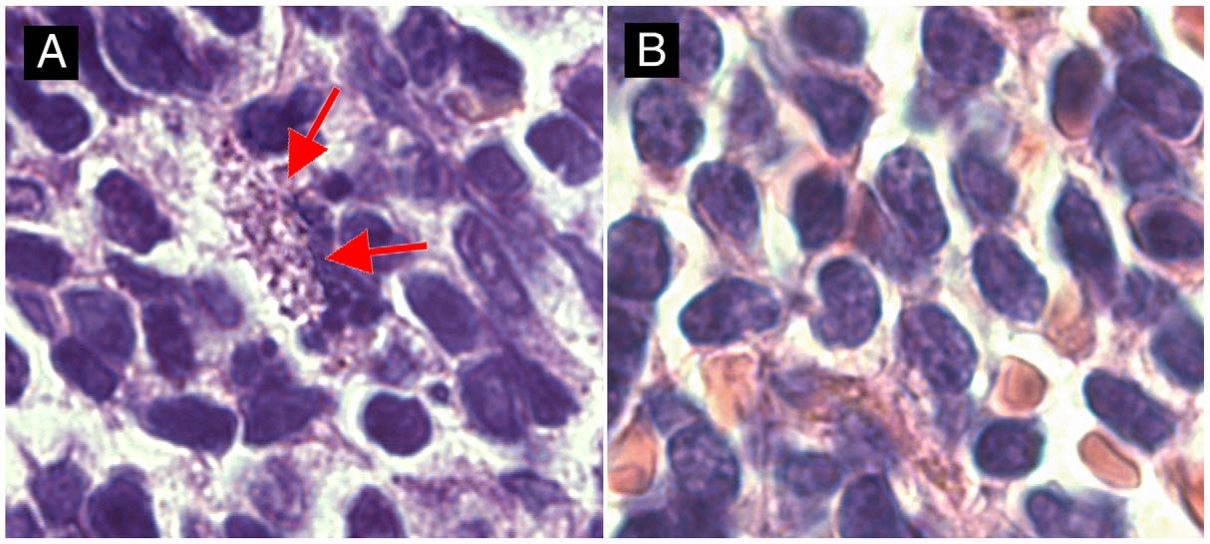
Parasites in hematoxylin-eosin stained spleen smears. Arrows show a group of parasites among spleen cells in infected BALB/c male (A); noninfected control BALB/c male without parasites (B).

We did not observe any splenomegaly and hepatomegaly induced by infection with *L. tropica*. However, *in vitro* cultures have shown that 21 weeks after infection 50% (3 out of 6), 66.7% (2 out of 3) and 16.7% (1 out of 6) spleens of female mice of the strains CcS-3, -18 and -20, respectively, contained viable parasites. We were also able to cultivate parasites from 16.7% (1 out of 6) and 33.3% (2 out of 6) of spleens of males of the strains BALB/c and CcS-20, respectively. Parasite presence in spleens was confirmed by histological examination (Figure 5). Parasite numbers in spleens of other strains were either below the level of detection or absent. Mice of the strain CcS-12 were not tested in the 21-week experiment.

Later we measured parasite load in frozen liver tissues using PCR-ELISA [27]. The presence of parasites after 21 weeks of infection was detected in females of the strains BALB/c 33.33% (2 out of 6), CcS-3 66.67% (4 out of 6), CcS-11 42.86% (3 out of 7), CcS-16 71.43% (5 out of 7), CcS-18 66.67% (2 out of 3) and CcS-20 75% (3 out of 4). Livers of males of the same strains also contained parasites: BALB/c 83.33% (5 out of 6), CcS-3 100% (3 out of 3), CcS-11 80% (4 out of 5), CcS-16 66.67% (4 out of 6), CcS-18 33% (2 out of 6) and CcS-20 83.33% (5 out of 6).

Unfortunately, an additional analysis of spleens and lymph nodes with PCR-ELISA cannot be performed as these organs were completely used for the cultivation assay.

### Systemic reactions after infection with *L. tropica*

We measured levels of IL-4, IL-12, IFNγ, GM-CSF (granulocyte-macrophage colony-stimulating factor), CCL2 (chemokine ligand 2)/MCP-1 (monocyte chemotactic protein-1), CCL3/MIP-1α (macrophage inflammatory protein-1α), CCL4/MIP-1β (macrophage inflammatory protein-1β), CCL5/RANTES (regulated upon activation, normal T-cell expressed, and secreted), CCL7/MCP-3 (monocyte chemotactic protein-3) and IgE in serum of uninfected and infected mice.

**Figure 6:**
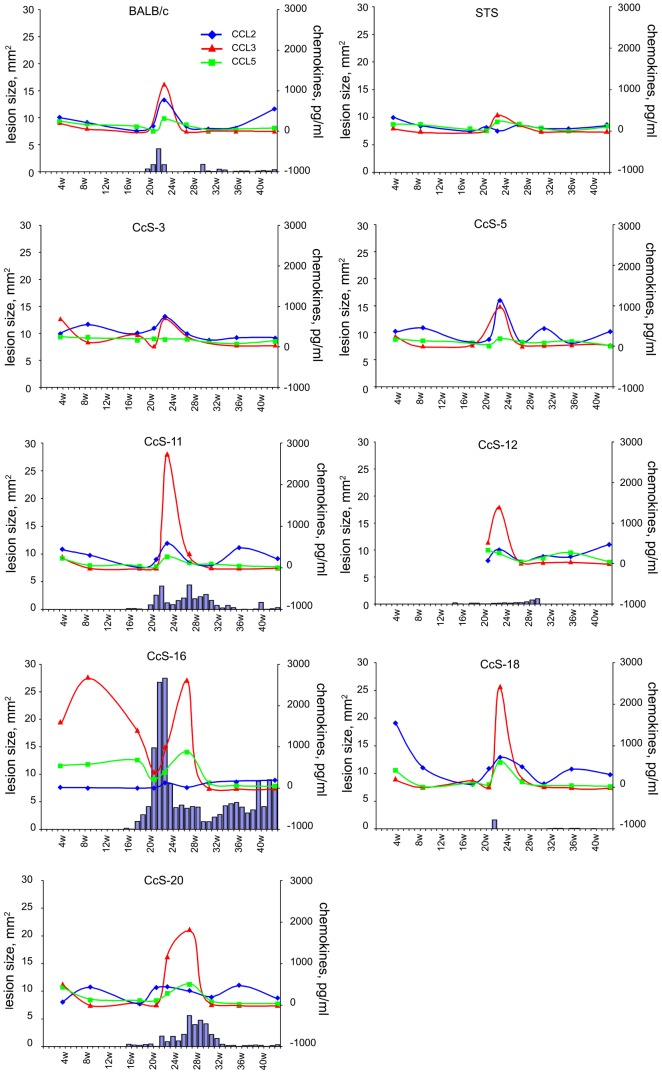
Relationship between chemokine expression and lesion size development during the course of *L. tropica* infection. Kinetics of skin lesion development (median, left y axis) and serum levels of CCL2, CCL3 and CCL5 (median values; right y axis) in females are shown. Figure summarizes data from two independent experiments (21 and 43 weeks of infection).

**Figure 7:**
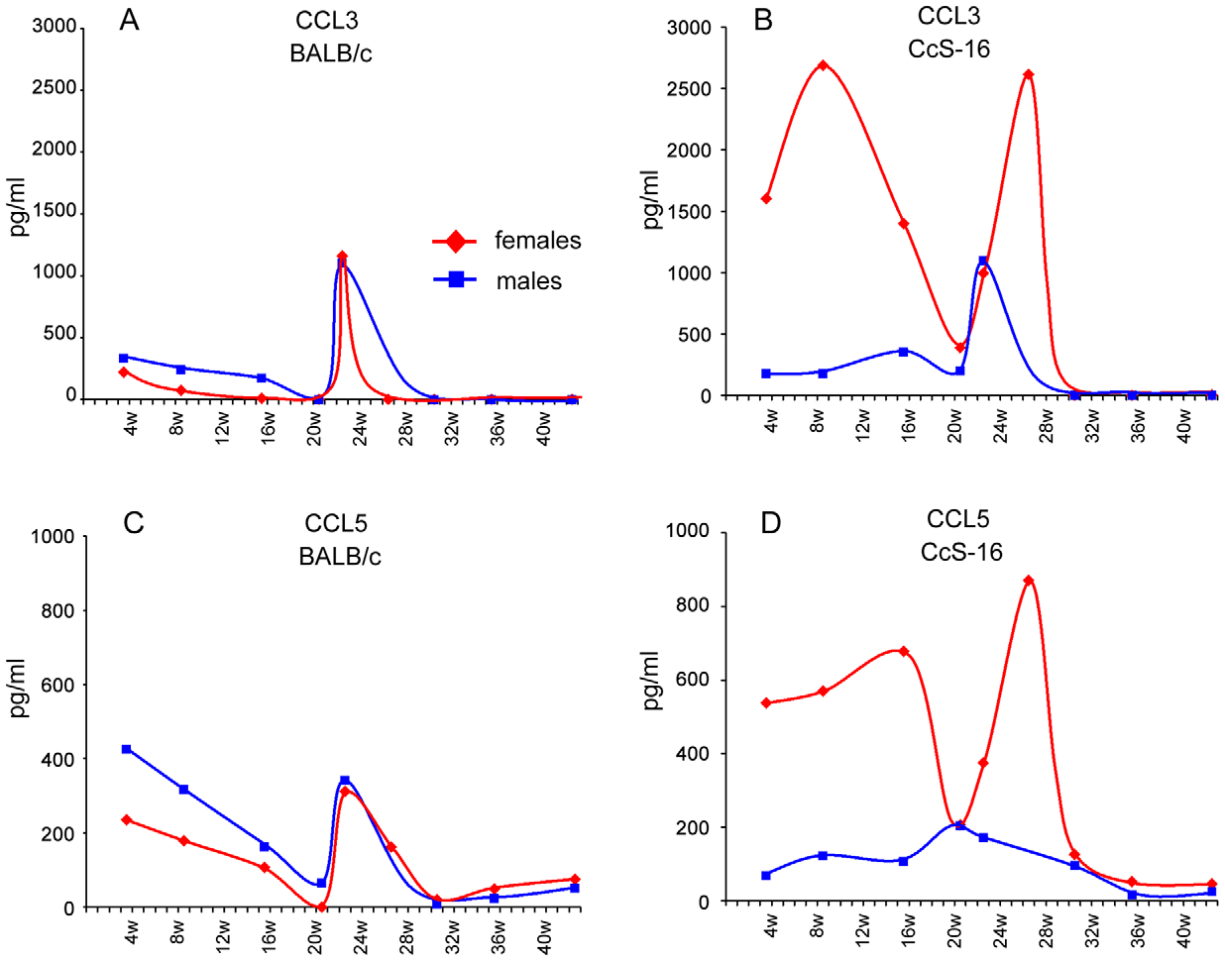
Comparison of kinetics of serum level of CCL3 and CCL5 in females and males of strains CcS-16 and BALB/c. Kinetics of CCL3 levels (median values) in serum of BALB/c (A) and CcS-16 (B) and serum levels of CCL5 (median values) in BALB/c (C) and CcS-16 (D) mice is shown. Figure summarizes data from two independent experiments (21 and 43 weeks of infection).

No significant difference was found in IL-4, IL-12, IgE, IFNγ and GM-CSF levels in serum of infected mice in comparison with noninfected controls (data not shown). We have observed an increase of serum levels of CCL2, CCL3 and CCL5 in infected strains; the largest increase was observed in strains CcS-11, CcS-16, CcS-18 and CcS-20. Figure 6 shows chemokine kinetics in females. Peak of increase of levels of these chemokines usually followed the start of lesion development. The increase was greater in females than in males (data not shown). Females of the strain CcS-16 exhibited a unique pattern of kinetics of CCL3 and CCL5 levels, which differed from all other strains (Figure 6) and also from CcS-16 males (Figure 7). We observed two peaks of increase of serum levels of CCL3 and CCL5 in females of CcS-16 (Figure 6); one before the development of skin lesions, the other after the decrease of lesion size, and there were almost no changes in CCL2 level. CcS-16 males had slight increase of CCL3, CCL5 (Figure 7), and CCL2 (data not shown); their kinetics of increase was similar to those in females and males of other strains.

## Discussion

We have analyzed manifestations of the disease and the immunological parameters, which constitute the pathology of leishmaniasis in mice (organ pathology, parasite load in organs, systemic immune response) after infection with *L. tropica*. Compared with infection with *L. major*, infection progressed more slowly, some manifestations (skin lesions, parasite load in organs) were less pronounced, some (splenomegaly, hepatomegaly) were absent in the tested strains, and the systemic immune response also differed. These observations reveal new areas where the comparative research of infection by the two species can contribute to a deeper genetic and functional understanding of responses to both and perhaps eventually lead to a common conceptual framework for their interpretation. Such more general scheme could not be readily obtained by analysis of any of them alone. A progress in the heavily under-investigated biology of responses to *L. tropica* infection may also have a translational potential, which may be significant in view of the recent wider recognition of the etiological role of this parasite in human disease. The areas highlighted by our results include the likely species specificity of at least some susceptibility genes, the dramatically different effects of sex on the response, and a different regulation of some systemic responses.

### Genotype effects are different in *L. tropica* and *L. major* infection

We have observed clearly different patterns in strains' susceptibility to *L. tropica* and *L. major*. Out of the nine strains tested, four (BALB/c, STS, CcS-5, CcS-16) exhibited similar relative susceptibility, whereas susceptibility of five strains (CcS-3, CcS-11, CcS-12, CcS-18 and CcS-20) to these two parasites differed. Strains CcS-3 and CcS-20 are resistant and susceptible, respectively, to development of skin lesions after infection with *L. tropica*, but intermediate to infection with *L. major*. The strains CcS-12, CcS-18, and CcS-16 are among the most susceptible to *L. major* infection [28], (Table 1), whereas with *L. tropica* CcS-12 and CcS-18 are clearly less susceptible than BALB/c and CcS-16 (Figure 2). The mice of strain CcS-11 are intermediate after infection with *L. major*, but after infection with *L. tropica* they died with small or no lesions, low parasite load in lymph nodes and with no detectable parasites in spleens. The cause of mortality of CcS-11 was not revealed by standard histo-pathological investigation. These differences indicate presence of species-specific susceptibility genes. Such genes were indicated also by results of Anderson and coworkers [16] who found that strains BALB/c and C57BL/6 had similar numbers of parasites in ear dermis and exhibited similar ear lesion development after infection with *L. tropica.* In contrast, these two strains differ greatly in susceptibility to *L. major* [29], (reviewed in [7] and [15]). Poly-specific response genes that control susceptibility to both *L. major* and *L. tropica* probably also exist, as the strains STS and CcS-5 are resistant and BALB/c and CcS-16 are susceptible to cutaneous disease induced by both parasite species (Figure 2, Table 1).

These data complement information about species-specific and poly-specific control of infection to *L. donovani*, *L. infantum*, *L. mexicana* and *L. major* (reviewed in [7]). Poly-specific and species-specific susceptibility genes are not limited to leishmaniasis, but were also indicated in susceptibility to other pathogens such as *Plasmodium* (reviewed in [30]). The data on susceptibility to *L. tropica* and *L. major* reported here is relevant for investigators of other species-specific responses.

Females of the strain CcS-16 that contains a set of approximately 12.5% genes of the donor strain STS and 87.5% genes of the background strain BALB/c exhibited the largest skin pathology (Figure 1, Figure 2A), exceeding skin manifestations in both parental strains BALB/c and STS. The observations of progeny having a phenotype, which is beyond the range of the phenotype of its parents, are not rare in traits controlled by multiple genes. It was observed in different tests of immune responses of RC strains *in vitro* [31]–[35] and *in vivo* [28], [36]. Similarly, analysis of gene expression from livers in chromosome substitution strains revealed that only 438 out of 4209 expression QTLs were inside the parental range [37]. These observations are due to multiple gene-gene interactions of QTLs, which in new combinations of these genes in RC or chromosomal substitution strains can lead to the appearance of new phenotypes that exceed their range in parental strains. Alternatively, with traits controlled by multiple loci, parental strains often contain susceptible alleles at some of them and resistant at others, and some progeny may receive predominantly susceptible alleles from both parents. However, we cannot exclude the possibility that the unique phenotype of CcS-16 may be caused by a spontaneous mutation, which had appeared during the inbreeding, similarly as for example a loss-of-function mutation in pyruvate kinase protecting RC strains AcB55 and AcB61 against malaria, which is absent in both parental strains A/J and C57BL/6 [38].

The strains CcS-3 and CcS-5, which are resistant to *L. tropica* share common STS-derived segments on chromosome 5 (a small part near the position 131.01 Mb); chromosome 6 (segment 32- 44 Mb); chromosome 8 (0–14.72 Mb) and on chromosome 10 (114.44–125.42 Mb)([23] and unpublished data). Interestingly, the segment on chromosome 10 overlaps with *Lmr5*, which controls resistance to *L. major* [19].

### Strong sex-genotype interaction after infection with *L. tropica*

Three phenomena related to sex influence on *Leishmania* infection deserve attention: i) a different sex influence on overall susceptibility to skin pathology after infection with relatively closely related pathogen species *L. tropica* and *L. major* (Figure 2, Table 1), ii) different sex influence on strains' susceptibility to development of skin lesions (Figure 2) and on parasite numbers in lymph nodes (Figure 3), and iii) sex influence on chemokine levels in serum (Figure 7).

In contrast to *L. major* infection in CcS/Dem RC strains where males exhibited either higher or similar pathology as females, in *L. tropica* experiments females were more susceptible to skin pathology than males. However, lymph nodes of females and males of most RC strains do not differ in *L. tropica* parasite load. The only exceptions are lymph nodes of the strains CcS-5 (and possibly STS), where males exhibit higher numbers of parasites than females and strain CcS-18, where females exhibit higher number of parasites than males (Figure 3). We have also observed a unique transient early peak of serum level of CCL3 and CCL5 in CcS-16 females, but not in CcS-16 males nor in any other strain (see the following section). These data suggest that some genes controlling susceptibility to *L. tropica* might be sex dependent or alternatively that this sex influence depends on genotype.

Different sex influence on susceptibility to *L. mexicana* and *L. major* was observed in DBA/2 mice where females were highly resistant and males susceptible to lesion development after infection with *L. mexicana*. On the contrary, although both female and male mice developed ulcerating lesions after infection with *L. major*, lesions healed in males but not in females [39]. Sex influenced liver parasite burdens after intravenous inoculation of *L. major* in strains BALB/cAnPt, DBA/2N and DBA/2J, males having higher parasite load than females [40].

Genotype influence on sex differences was described in studies of *L. major* infection [22], [41]. No sex differences in susceptibility were observed in BALB/cJ mice, whereas male B10.129(10 M)ScSn mice were relatively resistant to cutaneous disease, while females developed non-healing ulcerative lesions followed by parasites' metastases and death [41]

Comparison of *L. major* susceptibility in two strains, BALB/cHeA and CcS-11, has shown that there is no significant sex influence on skin lesion development, splenomegaly and hepatomegaly in these strains. Parasite numbers in lymph nodes in males of both strains were higher than in females; however in spleens only CcS-11 but not BALB/c males had higher numbers than females. These observations show that sex affects pathology of various organs differently and that this influence is modified by the host genotype [22].

These results indicate that data obtained with *L. tropica* (different sex influence on susceptibility to two relatively closely related pathogen species, sex and genotype interaction, and different sex influence on pathology in different organs) reflect a more general phenomenon. Other clear sex biases in incidence of disease, parasite burden, pathology, mortality, and immunological response against various parasites, have been observed in humans and in rodents (reviewed in [42]).

### Systemic reactions and a unique pattern of chemokine response in CcS-16 females, but not males of CcS-16

No significant difference was found in IL-4, IL-12, IFNγ and GM-CSF levels in serum of infected mice in comparison with noninfected controls (data not shown). This differs from increase of serum levels of IL-4, IFNγ and IL-12 observed in CcS/Dem strains after 8 weeks of infection [28]. Loci controlling serum levels of IL-4, IFNγ, IL-12, TNFα and IL-6 after 8 weeks of *L. major* infection are described in [19], [21], [22]. Similarly as after *L. tropica* infection, no increase was observed in serum GM-CSF level after infection with *L. major* (data not shown).

However, the absence of differences in serum levels of IL-4, IL-12, IFNγ and GM-CSF after infection does not exclude the possibility that they are involved in the local response to *L. tropica*. To test this alternative future experiments are needed, similar to those performed to establish the role of *Fli1* (Friend leukemia integration 1) in *L. major* infection model [43].

Infection with *L. tropica* led to increased serum levels of chemokines CCL2, CCL3 and CCL5. The highest increase was observed in the strains CcS-11, CcS-16, CcS-18, and CcS-20 (Figure 6, chemokine kinetics in females). The most prominent was the increase of CCL3/MIP-1α. Unexpectedly and in contrast with the other strains tested, the CcS-16 females but not males exhibited a unique pattern of this systemic reaction, characterized by an additional early peak of chemokine levels before the onset of cutaneous disease. It suggests that these early peaks of CCL3 and CCL5 (Figure 7) might be associated with an increased susceptibility of CcS-16 females to *L. tropica*. However, they could also reflect a stronger, but ineffective response.

CCL3 is produced by a range of cell types, including monocytes/macrophages, lymphocytes, mast cells, basophils, epithelial cells, and fibroblasts. Similarly, expression of CCL5 can be induced in activated T cells, macrophages, fibroblasts, epithelial and endothelial cells, and mesanglial cells [44], [45]. Although chemokines evolved to benefit the host, inappropriate regulation or utilization of these proteins can contribute to many diseases [45].

Both CCL3 and CCL5 bind to receptors CCR1, CCR5 and to chemokine decoy receptor D6. CCL5 also binds to CCR3 [45]. Genes *ccl3* and *ccl5* are situated on mouse chromosome 11; genes *ccr1*, *ccr5* and *ccbp2* (D6) are located on mouse chromosome 9 (Table S1). In CcS-16 *ccl5* is on a STS-derived segment, whereas the strain of origin of *ccl3* is not yet known; *ccr1*, *ccr5* and *ccbp2* are on BALB/c-derived segment ([23] and unpublished data).

The role of CC-chemokines CCL2, CCL3 and CCL5 in leishmaniasis has been tested in a number of studies (reviewed in [46], [47]). CCL2 and CCL3 stimulate anti-leishmania response via the induction of NO-mediated regulatory mechanisms to control the intracellular growth and multiplication of *L. donovani* [48]. CCL2 together with CCL3 also significantly enhanced parasite killing in *L. infantum* infected human macrophages [49]. In analysis of susceptibility to *L. major* in mouse, CCL5 contributed to host resistance, but CCL2 alone did not correlate with resistance [50]. In humans, CCL2 expression correlated with self healing cutaneous lesions, whereas CCL3 was associated with lesions of chronic progressive diffuse cutaneous leishmaniasis caused by *L. mexicana* [51]. These studies indicate that a coordinated interaction of several chemokines is important for successful immune response against *Leishmania*, but also that the role of different chemokines in defense against various *Leishmania* ssp. might differ. The observed strain differences and the double peak of CCL3 and CCL5 in CcS-16 females provide a novel potential starting point for investigation of the impact of inter-individual differences in chemokine response on pathogenesis of leishmaniasis.

### *L. tropica* parasites are present in lymph nodes of all strains, but in much lower numbers than in mice infected with *L. major*

In spite of relatively limited pathological symptoms, we found viable parasites in inguinal lymph nodes of all tested strains (Figure 3, 4). In some strains (CcS-3, -18, -20 females, and CcS-20 and BALB/c males) we also observed visceralization of parasites in the spleen (Figure 5); and in females and males of BALB/c, CcS-3, CcS-11, CcS-16, CcS-18 and CcS-20 we detected parasites in the liver. Similarly as in previous *L. major* experiments, which mapped genes controlling parasite numbers and demonstrated their distinctness from susceptibility genes [22], our present *L. tropica* studies confirmed that the extent of the pathological changes in different organs did not directly correlate with parasite load. This was especially obvious in CcS-3 mice, which were resistant to development of skin pathology, but nevertheless contained parasites in lymph nodes, spleen and liver. These data indicate that parasite spread to the different organs and other manifestations of the disease are dependent on the genome of the host. Absence of correlation between parasite load in organs or parasitemia and intensity of disease has been observed also after infection with several other pathogens such as *Toxoplasma gondii* [52], *Trypanosoma brucei brucei* [53], *Trypanosoma congolense* [54], *and Plasmodium berghei* [55].

In conclusion, the present observation that many of the RC strains tested with the two *Leishmania* species exhibited different susceptibility to *L. major* and *L. tropica* demonstrates existence of species-specific controlling host genes with different functions. Therefore, without combining the two components of variation involved in the outcome of *Leishmania* infection – genetic variation of the host and species of the parasite - the understanding of the mechanisms of disease will remain incomplete. On the basis of the observed strain differences we will perform linkage analysis of the responsible genes. This information may provide the first step to distinguishing the species-specific from the general genes controlling pathogenesis of leishmaniasis.

## Supporting Information

Figure S1 **Parasites in hematoxylin-eosin stained lymph node smears.** All tested mice contained viable parasites in their inguinal lymph nodes. Infected BALB/c female (A); noninfected control BALB/c female (B); infected BALB/c male (C); noninfected control BALB/c male (D); infected STS female (E); noninfected control STS female (F); infected STS male (G); noninfected control STS male (H). Parasites are shown with arrows.
(TIF)

Table S1 **ID numbers for genes of chemokines and cytokines.** ID numbers of genes, whose products were analyzed in this study, are shown.
(DOC)
