# Supplementary figures and images for: Genetics of Host Response to Leishmania tropica in Mice – Different Control of Skin Pathology, Chemokine Reaction, and Invasion into Spleen and Liver
Source: PLoS Negl Trop Dis. 2012 Jun 5;6(6):e1667. doi: 10.1371/journal.pntd.0001667 (PMC3367980; doi:10.1371/journal.pntd.0001667)

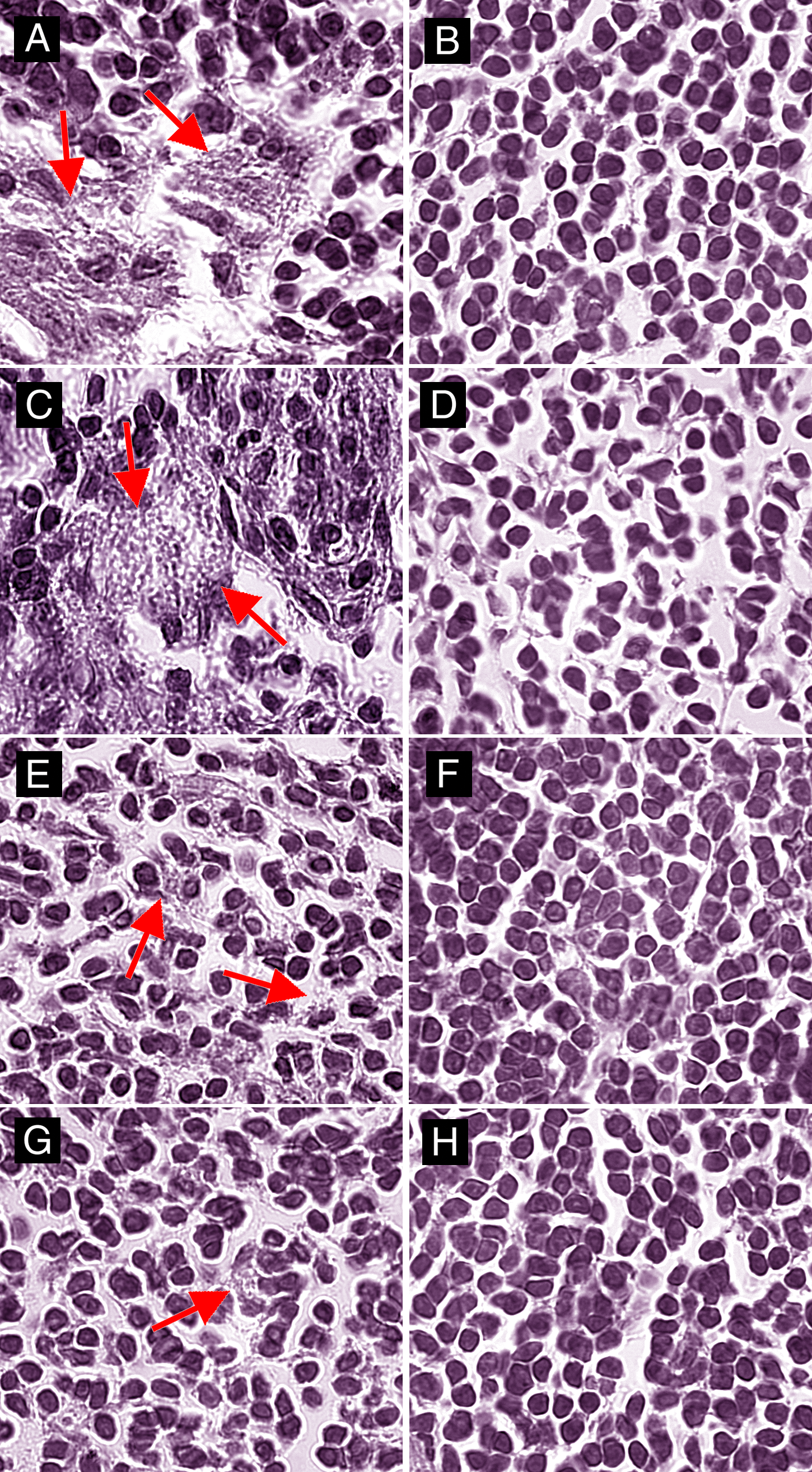

Supplement: Figure S1 — Parasites in hematoxylin-eosin stained lymph node smears. All tested mice contained viable parasites in their inguinal lymph nodes. Infected BALB/c female (A); noninfected control BALB/c female (B); infected BALB/c male (C); noninfected control BALB/c male (D); infected STS female (E); noninfected control STS female (F); infected STS male (G); noninfected control STS male (H). Parasites are shown with arrows. (TIF) [file pntd.0001667.s001.tif]
