# Supplementary material for: Genetics of Host Response to Leishmania tropica in Mice – Different Control of Skin Pathology, Chemokine Reaction, and Invasion into Spleen and Liver
Source: PLoS Negl Trop Dis. 2012 Jun 5;6(6):e1667. doi: 10.1371/journal.pntd.0001667 (PMC3367980; doi:10.1371/journal.pntd.0001667)
Supplement: Table S1 — ID numbers for genes of chemokines and cytokines. ID numbers of genes, whose products were analyzed in this study, are shown. (DOC) [file pntd.0001667.s002.doc]

**ID numbers for genes**

***Ccl2*** **chemokine (C-C motif) ligand 2** **[*Mus musculus* ]**

Gene ID: 20296

MGI: 98259

***Ccl3***

**chemokine (C-C motif) ligand 3 [*Mus musculus*]**

Gene ID: 20302

MGI: 98260

***Ccl5***

**chemokine (C-C motif) ligand 5 [*Mus musculus*]**

Gene ID: 20304,

MGI: 98262

**GM-CSF(*Csf2*)**

**colony stimulating factor 2 (granulocyte-macrophage) [*Mus musculus*]**

Gene ID: 12981

MGI: 1339752

***Ccl4***

**chemokine (C-C motif) ligand 4 [*Mus musculus*]**

Gene ID: 20303, updated on 26-Jan-2012

MGI: 98261

***Ccl7***

**chemokine (C-C motif) ligand 7 [*Mus musculus*]**

Gene ID: 20306, updated on 15-Jan-2012

MGI: 99512

***Ccr1***

**chemokine (C-C motif) receptor 1 [*Mus musculus*]**

Gene ID: 12768,

MGI: 104618

***Ccr5***

**chemokine (C-C motif) receptor 5 [*Mus musculus*]**

Gene ID: 12774

MGI: 107182

***Ccbp2***

**chemokine binding protein 2 [*Mus musculus*]**

Gene ID: 59289

MGI: 1891697

***Il12a***

**interleukin 12a [*Mus musculus* ]**

Gene ID: 16159

MGI: 96539

***Il12b***

**interleukin 12b [*Mus musculus*]**

Gene ID: 16160

MGI:96540

***Il4***

**interleukin 4 [*Mus musculus*]**

Gene ID: 16189

MGI: 96556

***Ifng***

**interferon gamma [*Mus musculus*]**

Gene ID: 15978

MGI: 107656
